# The economic burden of treating neonates in Intensive Care Units (ICUs) in Greece

**DOI:** 10.1186/1478-7547-5-9

**Published:** 2007-07-16

**Authors:** Mary Geitona, Magdalini Hatzikou, Zoi Hatzistamatiou, Aggeliki Anastasiadou, Theodora D Theodoratou

**Affiliations:** 1 Department of Economics, University of Thessaly, Volos, Greece; 2 Department of Neonatal Intensive Care Unit, Alexandra Hospital, Athens, Greece; 3 Department of Health Care Affairs, Boehringer Ingelhmeim Hellas, Ellinikou 2, Elliniko, P.C. 16777, Athens, Greece

## Abstract

**Background:**

In a period when a public-private mix in Greece is under consideration and hospital budgets become restrained, economic assessment is important for rational decision making. The study aimed to estimate the hospitalization cost of neonates admitted to the ICUs and demonstrate discrepancies with reimbursement.

**Methods:**

Chosen methodology was based on the selection of medical records of all NICUs and intermediate care admissions within February to April 2004. Neonates (n = 99) were classified according to birthweight and gestational age.

**Results:**

Mean cost per infant was estimated at €5.485 while reimbursement from social funds arises to €3.952. Costs per birthweight or gestational age show an inverse relationship. Personnel costs accounted for 59.9%, followed by enteral/parenteral feeding (16.14%) and pharmaceuticals expenses (11.10%) of all resources consumed. Sensitivity analysis increases the robustness of the results

**Conclusion:**

Neonatal intensive care in Greece is associated with significant costs that exceed reimbursement from social funds. Reimbursement should be adjusted to make neonatal intensive care economically viable to private hospitals and thus, increase capacity of the services provided.

## Background

The National Health System (ESY) in Greece was established in 1983 providing free access to all public services at the point of use. Given that the private sector had limited space for growth, it embarked on providing services in order to increase market share by setting up diagnostic centres and investing in expensive medical technology. As such, the Greek health care sector was converted in a mixed public-private system [1]. In 2004, the total healthcare expenditure in the country was 7.9% of the GDP of which 52.8% comes from the public sector while the remaining from the private sector [2].

Intensive care cost is one of the largest components of inpatient care worldwide ranging from 15% to 35% of hospital budgets and accounting for about 0.2% to 1.5% of gross domestic product (GDP) [3-7]. Neonatal intensive care for low birth weight infants is ranked among the most costly hospital admissions and is regarded as one of the most expensive components of pediatric health care accounting for about 10% of total pediatric expenses [7,8].

Utilization of NICUs has generated a great deal of concern in several countries due to their continuing increasing demand and resource consumption. Fast technological innovations and improved obstetric practices, in combination with the highly specialized personnel and the intense working conditions, are in large part responsible for the remarkable decline in neonatal and perinatal mortality rates and the aforementioned high share costs. In the international literature, intensive care has been proved cost-effective since the benefits for saving children's lives are far greater than the relative costs [3,8-11]. Hence, in an era of increased financial scrutiny and competing demands for limited healthcare resources, technology assessment constitutes a useful managerial tool since it enables decision-makers or health professionals to make more rational and cost-conscious clinical decisions.

In Greece, during the last decades there has been a significant reduction in neonatal (50%) and perinatal (64%) mortality rates reaching at 8‰ and 4‰ respectively. However, an increasing rate in the preterm birth and low birth weight newborns has been observed [12-14]. In addition, the continuing urbanization and depopulation of rural areas, gave a shift in the health services demand to the urban areas resulting to large socio-economic and geographical inequalities in the health services' provision [6,15,16]. In Greece there is an inadequate supply and unequal distribution of the NICUs beds in the various geographical regions. As presented in Table 1, the large urban areas (Athens and Thessaloniki) have the highest share of births as well as the majority of NICUs beds since they cover the majority of NICUs admissions and consequently, the lowest ratio of births per NICU beds.

**Table 1:** Distribution of NICU beds in public hospitals in Greece

**Geographical regions**	**Number of Births**	Beds (NICUs) *	**Births per NICU beds**
Greater Athens (Attiki)	14,851	85	175
Thessaloniki	6,660	30	222
Macedonia & Thrace	4,064	5	813
Western Macedonia	1,380	-	-
Western Greece	3,664	10	366
Ipiros	2,523	6	421
Ionian Islands	1,033	-	-
Peloponnesus	2,632	-	-
Crete	3,919	15	261
Aegean Islands	2,787	-	-
Thessaly	3,022	8	378
Central Greece & Evia	1,903	-	-

**Total**	**48,438**	**159**	**305**

Source: National Statistics Service of Greece, 2004

*The number of beds was given on request by the administration of the hospitals; the only official national reporting is the number of NICUS

Having identified the insufficient coverage of ICU beds of the public sector, the NHS introduced a public-private contract services system, which could allow reimbursement of the provision of intensive care for patients admitted to private hospitals so as to meet population needs. The creation of a public-private mix in the provision of intensive care faces great difficulties in Greece. The vast differentiation between NHS and private sector prices prevents social insurance funds to reimburse the provision of health care of their insured population in the private sector. The maintenance of a per diem hospital reimbursement system by the insurance funds results in deficits of the hospital budgets and prevents the private health providers to accept any public-private mix arrangements [7]. In addition, the lack of hospital costs assessments either for diagnostic related groups or surgical operations, as well as the overall insufficiency of national costing data, assigned priority to the cost analysis of the intensive care units.

Given the experience outlined above, the objective of the study is the cost analysis of resource consumption and the cost estimation for neonates admitted to NICUs in Greece, either per birth weight or per gestational age classification. The identification of cost per infant can facilitate public-private contracts to expand access to neonatal intensive care, once the significant underpayment by the social fund is taken into consideration.

## Methods

"Alexandra" and "Helena Venizelou" were the selected hospitals of the study. The selection of the specific hospitals was based on the fact that they are acknowledged as the two leading public obstetric and maternity hospitals of Athens representing almost 45% of the total number of deliveries that took place in Athens, in 2004.

Both hospitals have been incorporated into the National Health System (ESY) since 1986, cover 80% of the NICUs admissions of the public obstetric hospitals of Athens and 40% of the total NICU admissions in the public and private obstetric hospitals in the Athens area.

The District Maternity Hospital "Helena Venizelou" provides tertiary healthcare services with 7,000 deliveries approximately per year and one NICU with 20 mechanical ventilators. It also has a capacity of 384 beds, a prenatal control unit, along with a milk Bank and a Maternal Breast-feeding department. The second University Maternity Hospital "Alexandra" is one of the largest specialist hospitals in Athens, with a capacity of 300 beds, one NICU with 12 mechanical ventilators and 6,500 deliveries per year. According to patient records, although both hospitals are in the center of Athens, they host deliveries from Central Greece, Evia, the islands and neighboring counties of the Peloponnesus region.

Data was prospectively collected for all NICU and intermediate care admissions of both hospitals within a three month period (February to April 2004). The sample included neonates who had been admitted to both ICUs as premature, low birth weight and those who needed intensive care support for various reasons. Both hospitals examined are maternity hospitals and do not undertake surgeries or any type of operations. In cases of acute events that require the latter, neonates are transferred in paediatric hospitals.

Once the data was collected, infants were categorized into groups according to their birthweight (gr <1000, 1001–1500 gr, 1501–2000 gr, 2001–2500 gr and 2501 < gr) and to their gestational age (wk < 24, 24 ≤ wk < 28, 28 ≤ wk < 32, 32 ≤ wk) [18,19]. Neonates that did not survive formed a separate group (deaths) in the analysis to avoid potential underestimation of the cost per infant.

The estimation of cost was performed using a "bottom up approach", which identifies all the resources directly employed for an intervention. It is commonly used when considering technologies with a large component of staff input or overheads where healthcare systems do not allocate costs to the intervention level, such as the intensive care unit [20]. In these circumstances and since no national data were available this microeconomic approach was chosen to increase consistency and transparency of our results. Cost was based on the analytical recording of a) the resource consumption of supplies, medication, laboratory and medical tests and enteral and parenteral feeding on an everyday basis per infant, b) the infrastructure and various overhead costs such as electricity, cleaning, telephone, watering, heating, maintenance and repairs and c) personnel cost.

Public sector prices and NHS perspective have been used in the analysis. Depreciation of the capital assets was not included due to lack of data. For that reason, the analysis focused only on variable costs [21].

All personnel cost data were obtained from budgetary control statements provided by both hospitals' finance departments. Personnel cost includes the wages of the medical and nursing staff that was fully employed in the NICU and the intermediate section II of the two hospitals. It also includes the cost of paramedical, administrative and other personnel that were calculated based on the number of patients admitted for each hospital.

The cost of enteral and parenteral feeding was estimated based on the substances used for each infant. Cost of consumables was also reported on a per infant basis and was obtained by each hospital supplies department '[See Additional file 1]'. Infrastructure and general overhead costs such as electricity, water, heating, telephone and other utilities, were allocated based on the area occupied (square meters) by the NICU and the intermediate section II unit over the total area of the hospital. The cost of diagnostic tests, drug utilization and any other medical exams was reported on a per-case basis '[see Additional file 2]'.

The estimated cost was compared with the reimbursement from the social security funds based on the per diem payment (Government Gazette 99B/10-2-98). The amount reimbursed per inpatient day for the intensive care unit is at €187, while for the intermediate section II reaches €93.

From the data collected a descriptive statistical analysis was conducted. Mean ± standard deviation values are given when necessary and 95% confidence intervals are estimated. Sensitivity analysis was undertaken to explore the reliability of our estimates. The monetary values used in the paper have been converted in US Dollars in 2004 using an inflation rate of 3% for all studies to allow international comparisons. The conversion rates used depending on the study were:

January 1998: 1 US$: 0.61 GBP

January 2004: 1 US$: 0.79€

## Results

The study sample consisted of 99 neonates corresponding to approximately 45% of overall NICU admissions annually, 44 were from "Helena Venizelou" hospital and 55 from "Alexandra" hospital Table 2 presents the characteristics of the sample from both hospitals and the classification by birth weight and gestational age separately.

**Table 2:** Characteristics of the Sample (n = 99)

**Infants' Characteristics**	**In absolute numbers (%)**	
	
	**ELENA VENIZELOU HOSPITAL**	**ALEXANDRA HOSPITAL**
**No. of patients**	44 (44.4)	55 (55.5)
Male	28 (63.6)	30 (54.5)
Female	16 (36.4)	25 (45.5)
**Place of residence**		
Urban Areas	35 (79.5)	35 (63.6)
Rural Area	9 (20.5)	20 (36.4)
Mother's Age*	30.6 ± 5.1	29.44 ± 6.5
Mother's Insurance coverage	41 (93.2)	42 (76.4)
Mean Gestational Age* (wks)	35.1 ± 3.9	32.1 ± 5.06
Mean Weight (grs)*	2327 ± 787.8	1665 ± 846.4
**Weight Classification (n)**		
< 1000 gr	-	9 (16.4)
1001 – 1500 gr	5 (11.4)	13 (23.6)
1501 – 2000 gr	7 (15.9)	14 (25.4)
2001 – 2500 gr	9 (20.4)	6 (10.9)
≥2500 grs	20 (45.4)	9 (16.4)
**Gestational Age (n)**		
< 24 weeks	-	-
24 ≤ wks < 28	2 (4.5)	6 (10.9)
28 ≤ wks < 32	3 (6.8)	15 (27.3)
≥32 wks	36 (81.8)	30 (54.5)
Deaths	3 (6.8)	4 (7.3)
Mean LOS in NICU	9.8 ± 15.5	18.4 ± 17.4
Mean LOS in Section II	8.9 ± 8.3	16.1 ± 13.2
Mean Length of Stay (LOS)	18.7	34.5

* Mean ± Standard Deviation

Table 3 provides information on the cost drivers of the ancillary services, including pharmaceutical, laboratory, consumables and enteral/parenteral feeding costs. Pharmaceutical costs include the costs of any drug used during infants' overall stay in the hospital. During the study period, approximately 70 pharmaceutical products were used in the aforementioned NICUs and intermediate sections II of both hospitals. These included antibiotics, surfactants and others. Laboratorial costs included tests such as CBC, blood gas analysis, ultrasounds, MRIs and other.

**Table 3:** Total ancillary services cost by weight and gestational age in Athens in 2004 Euros (rounded to the closest integer)

**Infants Classification**	**N**	**Pharmaceutical Cost (€)**	**Laboratory Cost (€)**	**Consumables Cost (€)**	**Enteral/Parenteral Feeding (€)**	**Total Ancillary Cost (€)**
**Weight**						
< 1000 gr	9	24,222	5,431	3,566	11,980	**45,199**
1001 – 1500 gr	18	24,361	14,584	7,057	36,150	**82,151**
1501 – 2000 gr	21	8,835	7,164	3,470	24,090	**43,559**
2001 – 2500 gr	15	463	3,181	1,107	7,440	**12,191**
≥2500 grs	29	2,798	5,588	2,290	12,140	**22,816**
deaths	7	3,531	2,455	597	1,560	**8,143**

**Gestational Age**						

< 24 weeks	0	-	-	-	-	-
24 ≤ weeks < 28	8	11,146	7,730	3,722	17,560	**40,158**
28 ≤ weeks < 32	18	34,088	11,544	4,934	27,810	**78,377**
≥32 weeks	66	15,446	16,673	8,833	46,430	**87,382**
Deaths	7	3,531	2,455	597	1,560	**8,143**

**All Infants**	99	64,211	38,402	18,087	93,360	**214,060**

Table 4 presents analytical costs for infants' hospitalisation in NICUs in Athens. Mean cost per infant classified by birth weight and gestational age is presented. It is estimated at €5.845 [95% CI: 5.404, 6285] in 2004 with mean length of stay (LOS) of 27.5 days. It is observed that cost per infant is inversely related to both birth weight and gestational age which is compatible to the literature [9,13,14,19,22,23]. Also, the underpayment by social security funds becomes evident, except in the small for gestational age and very low birth weight infants were it is fully covered.

**Table 4:** Mean cost per infant in Athens in 2004 Euros (rounded to the closest integer)

**Infants Classification**	**N**	**Length of Stay (LOS)**	**Ancillary Cost (€)**	**Overhead Cost (€)**	**Cost of Personnel (€)**	**Total Cost (€)**	**Cost per Infant (€)**	**Amount Reimbursed (€)**
**Birth Weight**								
< 1000 gr	9	33.89	45,199	1,651	31,491	78,341	**8,705**	8,149
1001 – 1500 gr	18	34.50	82,151	3,302	62,981	148,434	**8,246**	8,441
1501 – 2000 gr	21	12.95	43,559	3,852	73,478	120,889	**5,757**	4,369
2001 – 2500 gr	15	5.3	12,191	2,751	52,484	67,427	**4,495**	1,941
≥2500 grs	29	5.0	22,818	5,319	101,470	129,607	4,469	1,380
deaths	7	3.9	8,143	1,284	24,493	33,919	**4,846**	724

**Gestational Age**								

< 24 weeks	0	-	-	-	-	-	**-**	
24 ≤ weeks < 28	8	55.9	40,158	1,467	27,992	69,617	**8,702**	8,757
28 ≤ weeks < 32	18	49.1	78,377	3,302	62,981	144,660	**8,037**	7,158
≥32 weeks	66	30.6	87,382	12,106	230,931	330,420	**5,066**	2,837
Deaths	7	3.9	8,143	1,284	24,493	33,919	**4,846**	724

**All Infants**	**99**	**27.5**	214,060	18,159	346,397	578,616	5,845*	**3,952**

* including deaths as well

According to the cost breakdown presented in Table 5, the highest share of resources was allocated in personnel wages (59.9%), in enteral/parenteral feeding (16.1%) and pharmaceuticals (11%).

**Table 5:** Cost breakdown per infant hospitalized in NICU (*rounded to the closest integer)

**Cost components**	Mean Cost per infant* in €	**Cost distribution in %**
Laboratory-diagnostic tests	388	6.64
Pharmaceutical expenses	649	11.1
Enteral/Parenteral costs	943	16.1
Consumables	183	3.1
Personnel Cost	3.499	59.9
Overhead Cost	183	3.1
**Total cost**	**5.845**	**100**

### Sensitivity analysis

From the data presented in table 4, one can observe that length of stay (LOS) is highly skewed. Therefore, a sensitivity analysis was run to correct for the non-normality of the distribution by excluding outliers (n = 10); all came from the lowest gestational and/or birthweight clasification (Table 6). The difference in mean cost per infant between the two scenarios shows statistical significance between the groups (p = 0,018). However, in everyday clinical practice neonatal intensive care unit admits extremely low birth weight infants accounting for 5–8% of the overall premature infants [18]. Thus, it was assumed appropriate not to treat them as outliers and exlude them from the analysis.

**Table 6:** Sensitivity Analysis Results

**Infants Classification**	**N**	**Length of Stay (LOS)**	**Ancillary Cost (€)**	**Cost of Infrastructure (€)**	**Cost of Personnel (€)**	**Total Cost (€)**	**Cost per Infant (€)**
**Birth Weight**							
< 1000 gr	5	31.8	18,909	1,020	19,460	39,390	**7,878**
1001 – 1500 gr	12	43.7	40,852	2,448	46,705	90,005	**7,500**
1501 – 2000 gr	21	33.6	43,559	4,285	81,734	129,578	**6,170**
2001 – 2500 gr	15	15.3	12,191	3,061	58,381	73,633	**4,909**
≥2500 grs	29	9.8	22,816	5,917	112,871	141,604	**4,883**
deaths	7	3.9	8,143	1,428	27,245	36,816	**5,259**

**Gestational Age**							
< 24 weeks	0	-	-	-	-	-	**-**
24 ≤ weeks < 28	8	30.5	11,819	816	15,568	28,204	**7,051**
28 ≤ weeks < 32	18	40.7	49,675	2,857	54,489	107,021	**7,644**
≥32 weeks	66	18.9	76,833	13,058	249,094	338,985	**5,297**
deaths	7	3.9	8,143	1,428	27,245	36,816	**5,259**

**All Infants**	**89**	**21,7**	146,470	18,159	346,397	511,026	**5,742**

In order to reflect better the cost of the selected neonates the median cost per infant was also used. It was estimated at €4.927 (Q1: 4,371; Q3: 6,604; IQ: 2,232), showing a difference of €918 from the mean.

## Discussion

The present cost assessment was arisen from the insufficient coverage of beds in the intensive care units in general and in particular for neonates, of the public hospitals in the country. The continuously rising demands for NICUs in the big urban centers, the lack of ICU beds and the unequal geographical distribution of public NICUs has recently lead the government to contract intensive care services from the private sector. Although the adoption of such a policy is thought to bring about positive results, so far its outcomes have not been evaluated.

The current study had as primary objective to estimate the cost of neonatal intensive care unit. The estimation of the hospitalization cost of neonates in comparison to the amount reimbursed from the social security funds could lead to a successful synergy between the public and private healthcare sector, to the reduction of public hospitals' deficits and to empower coverage of social security funds with the private hospitals.

According to the results of the study, the mean cost per infant hospitalized in a NICU in the two major public maternity hospitals in Athens, was estimated at €5.845, when the social security funds reimbursed €3.952 for every neonate admitted. This finding is important since it depicts the factors responsible for a potential failure of this public-private mix in the provision of intensive care for neonates. Private hospitals may not be willing to accept reimbursement according to the infants' nominal cost (€ 3.952). Reimbursement should be adjusted to make neonatal intensive care economically viable to private hospitals and thereby increase capacity of the service overall.

It is believed that the importance of carrying out such a study was threefold: firstly, because it relates to the analytical identification of cost drivers and the assessment of resource consumption per infant in a NICU in Greece. Secondly, it involved the estimation of hospitalisation costs according to patients' classification. Similar studies in Greece are not often encountered in the literature due to the lack of specialty unit prices based on diagnostic or clinical performance criteria [6,24]. Thirdly, because it becomes evident that cost analysis allows the possibility of introducing diagnostic related patients' grouping and abandoning the per diem payment, which results in enormous deficits in the public hospitals' budgets [15-17]. It is believed that the classification of infants, per birth weight or gestational age, could easily constitute diagnostic related patients' categories combined with a prospective hospital payment system. In that case, it is apparent that private health care providers would have some economic motivation in profiting through the public-private mix expansion in the country.

In an effort to compare our results to those of the international literature in cost analysis of NICUs, differences and similarities have been observed. Differences can be explained by the availability of economic data, the choice between private or public health care sector unit prices and the variability of the accounting methods for measuring the economic costs. According to an extensive literature review performed by Petrou [13] the mean cost per infant category of 1001–1500 gr, was $45,152 for studies conducted in the US and $19,975 for the non US studies (in 2004 USD). A study conducted by the Washington Office of Technology Assessment [8] estimated the mean cost per infant weighting 1501–2000 gr to be $10,948 and for those with birthweight over 2500 gr at $1,531. In the present study the mean cost per infant hospitalised in a public NICU was estimated at $10,438 for infants weighting 1001–1500 gr, while for those exceeding 2500 gr reached $5,657.

Furthermore, as reported earlier, it was found that infants' hospitalisation cost varies inversely with birth weight, since it reflects differences in both the mean length of stay and in the intensity of treatment during each day of stay [7,13,19,25]. In addition, the high share of the personnel, parenteral feeding and pharmaceutical care costs is an evident found in most cost analysis studies both in Greece and abroad [25-27].

At this point some methodological limitations should be mentioned. The mean cost per infant is underestimated primarily due to the use of NHS prices, which are far lower than those of the private maternity hospitals. The lack of data and the non-responsiveness of the private sector to provide the necessary information did not allow us to estimate the latter. Secondarily, due to the fact that the official registration of public hospitals' expenses did not include costs for depreciation of capital assets, so they were excluded from our microeconomic analysis.

To avoid overestimation of cost per infant in categories with small sample size, groups were collapsed to provide more reliable results. Additionally, sensitivity analysis increased the reliability of the results, since it showed that mean cost per infant were similar when excluding outliers (€5,845 vs. €5,742), despite the fact that the difference was found statistically significant.

## Conclusion

Through the analysis of this study it becomes evident that the identification of cost per infant can facilitate public-private contracts to expand access to neonatal intensive care, once the significant underpayment by the social fund is adjusted. It seems most important at this time period when a public – private mix effort is under consideration and hospital budgets become more restrained. For all the above-mentioned reasons, economic assessment is necessary in Greece since it will facilitate policy makers in their decision making.

## Authors' contributions

Professor Mary Geitona contributed to the conception and design of the study and both Magdalini Hatzikou and Theodora Theodoratou contributed to the analysis and interpretation of the data. All contributed equally to the writing of the manuscript. Finally, Dr. Zoi Hatzistamatiou and A. Anastasiadou were responsible for the acquisition and collection of the data from Alexandra Hospital and contributed to the paper by revising it critically. All authors have read and approved the final version of the manuscript.

## Supplementary Material

Additional file 1 Appendix 1 – Unit Costs of consumables. The data provided represent the unit costs and resource consumption of the consumables used during infants stay Click here for file

Additional file 2 Appendix 2 – Unit Costs of laboratory tests. The data provided represent the unit cost and frequencies of the different diagnostic imaging and laboratory exams undertaken. Click here for file
