# Supplementary material for: The economic burden of treating neonates in Intensive Care Units (ICUs) in Greece
Source: Cost Eff Resour Alloc. 2007 Jul 16;5:9. doi: 10.1186/1478-7547-5-9 (PMC1939832; doi:10.1186/1478-7547-5-9)
Supplement: Additional file 1 — Appendix 1 – Unit Costs of consumables. The data provided represent the unit costs and resource consumption of the consumables used during infants stay [file 1478-7547-5-9-S1.doc]

**APPENDIX 1 – UNIT COSTS FOR CONSUMABLES**

| **TYPE OF CONSUMABLE** | **FREQUENCY** | **PRICE** |
| --- | --- | --- |
| Umbilical Drainage Tube | 60 | 2,02 |
| Dextro Tapes | 5341 | 0,33 |
| Mucus aspiration | 1754 | 0,75 |
| Trocar | 1 | 5,03 |
| mask | 108 | 1,01 |
| 3-ways | 169 | 0,21 |
| Pamper | 23091 | 0,085 |
| Urine bags | 1794 | 0,09 |
| Bullaw Valves | 1 | 8,8 |
| Infant matrices | 4909 | 0,5 |
| Tubes for parenteral feeding (code 1155-10) | 353 | 1,76 |
| Artery tubes (code 1155-01) | 216 | 1,76 |
| Syringe | 29400 | 0,058 |
| Soluset | 241 | 1,59 |
| Red Dot 258-3 (monitor stickers) | 1423 | 0,69 |
| Feeding catheters | 2928 | 0,31 |
| Vene-catherers | 1803 | 0,79 |
| Respiratory circulators (single use) | 72 | 14 |
| Respiratory circulators (multiple use) | 12 | 14 |
| Scarificators | 2985 | 0,02 |
| Milk | 3473,5 | 0,3 |
| Dummies | 858 | 0,18 |
| Scalp-wen | 422 | 0,25 |
| Nellcor rheophores | 7 | 17 |
| Pharmaceutical provision through Raizer | 3 | 1,26 |
| Filiforms | 776 | 1,4 |
