# Supplementary material for: The economic burden of treating neonates in Intensive Care Units (ICUs) in Greece
Source: Cost Eff Resour Alloc. 2007 Jul 16;5:9. doi: 10.1186/1478-7547-5-9 (PMC1939832; doi:10.1186/1478-7547-5-9)
Supplement: Additional file 2 — Appendix 2 – Unit Costs of laboratory tests. The data provided represent the unit cost and frequencies of the different diagnostic imaging and laboratory exams undertaken. [file 1478-7547-5-9-S2.doc]

**APPENDIX 2 – UNIT COSTS OF LABORATORY TESTS**

| **TYPE OF EXAMINATION** | **FREQUENCY** | **UNIT PRICE (€)** |
| --- | --- | --- |
| Ca-P-Aφ | 425 | 4,05 |
| CoomBs | 89 | 2,88 |
| CRP | 365 | 13,21 |
| Electrolytes | 498 | 5,22 |
| K | 9 | 5,22 |
| Na | 6 | 5,22 |
| OMAS | 82 | 4,49 |
| RH | 83 | 4,49 |
| SGOT/ SGPT | 104 | 4,49 |
| Blood Gas | 782 | 16,02 |
| X-ray | 236 | 4,05 |
| CT scan / MRI | 3 | 71,11 |
| γGT | 71 | 5,02 |
| HDL/LDL | 1 | 4,75 |
| Cerebrospinal fluid | 34 | 4,05 |
| Coagulation Profile | 3 | 4,05 |
| Complete Blood Count | 735 | 2,88 |
| Glucose | 956 | 2,26 |
| Dextro-stick | 972 | 1,76 |
| Cerebrospinal fluid Culture | 35 | 5,22 |
| Urine culture | 25 | 5,22 |
| Blood culture | 345 | 5,22 |
| Omphalus Culture | 1 | 5,22 |
| Colon Culture | 8 | 5,22 |
| Creatinine | 263 | 4,05 |
| HIV test | 1 | 23,48 |
| Leukocytes | 24 | 2,88 |
| Urea | 497 | 2,26 |
| hemofycin | 6 | 9,51 |
| Theophyll | 11 | 9,75 |
| gathric test | 4 | 4,05 |
| vencomyre | 5 | 9,51 |
| Bilirubin | 597 | 2,88 |
| ***Miscellaneous diagnostic*** |  |  |
| Ophthalmology exam | 61 | 2,26 |
| Liver Ultrasound | 1 | 8,28 |
| Brain ultrasound | 119 | 8,28 |
